# Association of triglyceride-glucose index, low and high-density lipoprotein cholesterol with all-cause and cardiovascular disease mortality in generally Chinese elderly: a retrospective cohort study

**DOI:** 10.3389/fendo.2024.1422086

**Published:** 2024-10-29

**Authors:** Donghai Su, Zhantian An, Liyuan Chen, Xuejiao Chen, Wencan Wu, Yufang Cui, Yulin Cheng, Songhe Shi

**Affiliations:** ^1^ Department of Epidemiology and Health Statistics, College of Public Health, Zhengzhou University, Zhengzhou, Henan, China; ^2^ Department of Orthopedics, Hongxing Hospital, 13th Division, Xinjiang Production and Construction Corps, Hami, Xinjiang, China; ^3^ Department of Epidemiology and Health Statistics, College of Public Health and Management, Wenzhou Medical University, Wenzhou, Zhejiang, China

**Keywords:** triglyceride-glucose index, low density lipoprotein cholesterol, high density lipoprotein cholesterol, all-cause mortality, cardiovascular disease mortality

## Abstract

**Background:**

The impact of baseline triglyceride-glucose (TyG) index and abnormal low or high-density lipoprotein cholesterol (LDL-C or HDL-C) levels on all-cause and cardiovascular disease (CVD) mortality remains unclear. This study aimed to investigate the relationship between TyG index and LDL-C or HDL-C and all-cause and CVD mortality.

**Methods:**

This retrospective cohort study analyzed data from health examinations of 69,068 older adults aged ≥60 in Xinzheng City, Henan Province, China, between January 2013 and January 2023. Cox proportional risk regression models were used to estimate the hazard ratio (HR) and 95% confidence interval (CI) of the TyG index and LDL-C or HDL-C about all-cause and CVD mortality. Restricted cubic spline was used to assess the dose-response relationship.

**Results:**

During 400,094 person-years of follow-up (median follow-up 5.8 years [interquartile range 3.0-9.12]), 13,664 deaths were recorded, of which 7,045 were due to CVD. Compared with participants in the second quartile of the TyG index, participants in the fourth quartile had a 16% increased risk of all-cause mortality (HR: 1.16, 95% CI: 1.12,1.22), and an 8% increased risk of CVD mortality (HR: 1.08, 95% CI: 1.01,1.16). Similar results were observed in LDL-C and HDL-C, with all-cause and CVD mortality risks for participants in the fourth quartile compared with participants in the third quartile for LDL-C of (HR: 1.07, 95% CI: 1.02,1.12) and (HR: 1.09, 95% CI: 1.01,1.17), respectively. The risk of all-cause and CVD mortality in participants in the fourth quartile group compared with those in the second HDL-C quartile group was (HR: 1.10, 95% CI: 1.05,1.16) and (HR: 1.11, 95% CI: 1.04,1.18), respectively. We found that the TyG index was nonlinearly associated with all-cause and CVD mortality (P non-linear <0.05), and LDL-C was nonlinearly associated with all-cause mortality (P non-linear <0.05) but linearly associated with CVD mortality (P non-linear >0.05). HDL-C, on the other hand, was in contrast to LDL-C, which showed a non-linear association with CVD mortality. We did not observe a significant interaction between TyG index and LDL-C or HDL-C (P >0.05).

**Conclusion:**

TyG index and LDL-C or HDL-C increased the risk of all-cause and CVD mortality, especially a high TyG index combined with abnormal LDL-C.

## 1 Background

Cardiovascular diseases (CVD) are the major cause of death and premature mortality in China (1, 2). The burden of CVD continues to increase annually, with approximately 330 million CVD patients in China. CVD is attributable to 2 out of every 5 deaths in China (3). The Global Burden of Disease (GBD) Study reports that the total prevalence of CVD worldwide has increased from 271 million in 1990 to 523 million in 2019. Additionally, the number of deaths has increased from 12.1 million to 18.6 million, and this trend is continuing (4).

Insulin resistance (IR), physiologically defined as a state of reduced responsiveness of insulin-targeted tissues to high physiologic insulin levels, is recognized as a causative driver of many modern diseases, including metabolic syndrome (Mts), type 2 diabetes mellitus (T2DM), and CVD complications (5). The main factors contributing to the development of IR are increased oxidative stress, hyperglycemia, and elevated lipid levels (6). Although advances have been made in therapies to help control blood glucose levels, CVD complications remain a major cause of morbidity and mortality in this population (7). The Homeostasis Model Assessment of Insulin Resistance (HOMA-IR) is the most widely used surrogate indicator of IR but has limitations due to its complexity and cost, and it cannot be used in populations receiving insulin therapy (7). The triglyceride glucose (TyG) index is a low-cost and convenient tool for assessing IR in diabetic and non-diabetic patients (8, 9). Using HIEC and HOMA-IR as reference methods, the diagnostic accuracy of the TyG index in identifying IR has been tested in several studies. The highest sensitivity for HIEC was 96% and the highest specificity for HOMA-IR was 99% (8). The TyG index has also shown good performance in the estimation of IR in diabetic and non-diabetic patients compared to HOMA-IR (8). In addition, the TyG index does not require insulin quantification and can be used in all people, regardless of their insulin therapy status (9). However, fewer studies have been conducted on the association between the TyG index and CVD mortality.

IR not only makes individuals susceptible to CVD, it also enhances the effects of dyslipidemia (10). Dyslipidemia, mainly low or high-density lipoprotein cholesterol (LDL-C or HDL-C) in the abnormal range, is considered one of the major risk factors for CVD (11). Previous epidemiological studies have suggested that a higher TyG index is an important risk factor for all-cause and, in particular, CVD mortality (12, 13). However, these studies were all conducted in the general population. Furthermore, while a causal association between LDL-C and CVD mortality has been demonstrated (14), there are conflicting results regarding the relationship between HDL-C and CVD mortality. For example, some studies have suggested that higher HDL-C may be a better preventive factor for CVD (15), while others have found that high HDL-C levels are associated with an increased risk of CVD (16). Some studies have suggested a U-shaped pattern of all-cause and CVD mortality associated with LDL-C (17, 18), with some studies showing a linear relationship (19). It is also worth noting that there is limited research on the effect of the interaction between the TyG index and LDL-C or HDL-C on mortality risk.

To our knowledge, over the past 20-30 years, the health status of China’s total population has improved dramatically, with a significant increase in life expectancy, which has also meant a rapid and sustained increase in the aging population. Aging is considered to be an immutable factor that cannot be analyzed as a major influencing factor, so studies focusing on the elderly population are crucial. At the same time, the elderly are at high risk for CVD (20). Therefore, we wanted to explore the relationship between TyG index and LDL-C or HDL-C with all-cause and CVD mortality and to investigate the interaction between TyG index and LDL-C or HDL-C on risk in the elderly population.

## 2 Methods

### 2.1 Study design and population

**Figure 1:**
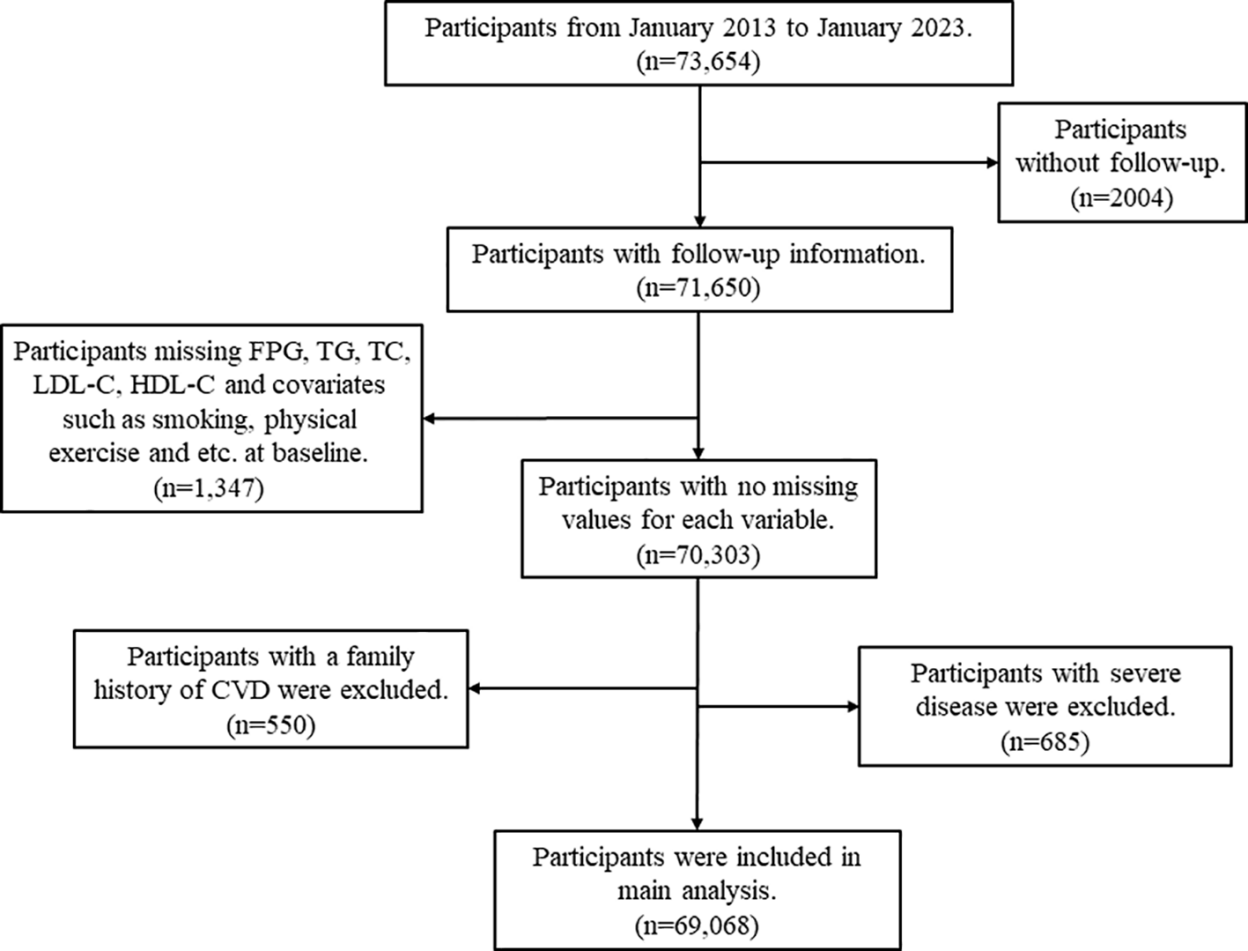
Flow chart of study participants.

The data analyzed were obtained from the Resident Health Examinations Database of Xinzheng City, Henan Province, Central China, which is a large-scale cohort study of older adults aged 60 years and older conducted by the Centers for Disease Control (CDC) and Prevention of Xinzheng City and contains sociodemographic and mortality information on the population of Xinzheng City. Since January 1, 2011, Xinzheng City has been providing free annual health examinations to senior citizens aged 60 and older. At the initial examination, the physician creates a health profile for each resident, which includes basic demographic information (age, gender, marital status, etc.), blood indicators (fasting plasma glucose (FPG), triglycerides (TG), total cholesterol (TC), etc.), urinalysis, eye examination, chest X-ray, and other functions. For this study, we obtained follow-up information from 2013 to 2023 from a total of 56,069 eligible older adults. The Framingham study revealed that premature development of CVD in first-degree relatives, such as parents or siblings, is associated with an elevated risk of subsequent development in the offspring. The presence of genetic factors may lead to the occurrence of heart disease, stroke, heart failure, and other serious illnesses (serious mental illness and cancer) in several family members (21). Consequently, individuals with a family history of CVD such as coronary heart disease (CHD), stroke, and myocardial infarction (MI) at the start of the study were excluded. Participants with any of the following were excluded: (1) Exclude participants with a family history of CVD (n=550); (2) Exclude those with missing FPG, TG, TC, LDL-C, and HDL-C at baseline (n=1066); (3) Exclude those with missing one or more of the covariates of smoking, alcohol consumption, physical activity, waist circumference (WC), body mass index (BMI) at baseline (n=281); (4) Exclude participants with serious illnesses, including serious mental illness and cancer (n=685); and (5) Exclude participants with no follow-up records (n=2004). The process of screening the data is presented in **Figure 1**.

### 2.2 Statement

The study was approved by the Ethics Committee of Zhengzhou University (ID: ZZUIRB2019-019), and the research team obtained permission to use the data from the Zhengzhou Health Commission. All studies were conducted by the Declaration of Helsinki, and informed consent was obtained from all participants or their legal guardians.

### 2.3 Data collection

Standardized questionnaires were administered by trained researchers, and participants completed a questionnaire on sociodemographic characteristics, personal disease history, and lifestyle information at each health examination. Sociodemographic information included participants’ age, gender (male/female), and marital status (married/unmarried/widowed/divorced); disease history information included hypertension (yes/no), T2DM (yes/no), CHD (yes/no), stroke (yes/no) and cancer (yes/no). Lifestyle information included smoking status (never/ever/current), drinking status (never/occasional/more than once a week/daily), and physical activity status (never/occasional/more than once a week/daily). Current smoking was defined as having smoked more than 100 cigarettes in a lifetime and currently smoking (22). Alcohol consumption is defined as drinking more than 30 grams of alcohol in a single sitting, and more than 30 gram of alcohol per day is considered to be alcohol consumption for the day (23). Regular exercise is defined as 30 minutes of moderate-intensity exercise or 20 minutes of vigorous-intensity exercise three or more times per week (24). Participants’ height, weight, blood lipids, WC, FPG, systolic blood pressure (SBP), diastolic blood pressure (DBP), and resting heart rate (RHR) were measured by trained health professionals. Blood samples taken after participants fasted for 8 hours were used to measure FPG and blood lipids. Blood pressure (BP) was measured using an electronic sphygmomanometer (Omron HEM-7125, Kyoto, Japan). The subjects were instructed to rest quietly for five minutes in a standard supine position. Two measurements of SBP and DBP were then taken in the right brachial artery, with an interval of 30 minutes between each measurement. The average level was taken as the result of the BP measurements. Hypertension was defined as SBP ≥140 mmHg and DBP ≥90 mmHg or the use of antihypertensive medication (25). T2DM was defined as FPG ≥7.0 mmol/L or use of insulin or oral hypoglycemic agents, or a self-reported history of T2DM diagnosis (26). BMI was calculated as weight (kilograms) divided by the square of height (meters). A scoring scale consistent with Chinese body mass was used. The TyG index, calculated as TyG index = ln [Fasting TG (mg/dl) × FPG (mg/dl)]/2, is a composite indicator composed of TG and FPG levels (27). The diagnostic criteria for abnormal LDL-C is a level of greater than 130 mg/dl, and for HDL-C, a level of less than 40 mg/dl for men or less than 50 mg/dl for women is considered abnormal (28).

### 2.4 Outcomes

The outcome of interest was all-cause and CVD mortality. We defined CVD as a composite of CHD and stroke. Mortality causes were recorded using the international Classification of Diseases (ICD-10) codes. The study utilized ICD-10 codes I20-I25 for CHD and ICD-10 codes I60-I69 for stroke. All-cause mortality was defined as deaths resulting from any cause, while CVD mortality was defined as deaths resulting from either CHD or stroke.

### 2.5 Statistical analysis

Baseline characteristics of participants were presented based on their grouping by all-cause or CVD mortality, and the Kolmogorov-Smirnov test was used to verify the normal distribution of the data. Continuous variables that followed a normal distribution were described as mean (standard deviation), while non-normal variables were described as median (interquartile range). Descriptive variables were presented as frequencies and percentages. To compare baseline characteristics, categorical variables were analyzed using the Pearson chi-square test and continuous variables were analyzed using the Kruskal-Wallis H test.

Cox proportional risk regression models were used to estimate the hazard ratio (HR) and 95% confidence interval (CI) of the TyG index, LDL-C, and HDL-C for all-cause or CVD mortality. Model 1 was not adjusted, while model 2 was adjusted for age and gender at baseline. Finally, model 3 was adjusted for marital status, smoking, alcohol consumption, physical activity, SBP, DBP, BMI, WC, history of T2DM, and history of hypertension, based on model 2.

Restricted cubic spline plots were used to characterize the dose-response associations and to examine potential linear or non-linear associations between the TyG index, LDL-C, and HDL-C as continuous variables, and all-cause or CVD mortality. The three nodes of the cubic spline curve were set at the 10th, 50th, and 90th percentiles, respectively. The overall association was initially assessed for significance, and if significant, the results of the linear and non-linear tests were examined. A significance level of *P <*0.05 was reached for both the overall association test, which indicated that the overall association was significant, and a non-linear level of *P <*0.05, which indicated the presence of a non-linear association.

Thresholds were estimated by testing all possible values and selecting the threshold point with the highest likelihood. Additionally, a two-segment Cox proportional risk model was used to examine the relationship between TyG index, LDL-C or HDL-C, and the risk of all-cause or CVD mortality on both sides of the inflection point.

In subgroup analyses, participants were stratified based on gender (male/female), and age (<65/≥65) at baseline to test for differences in outcomes across subgroups. To test the robustness of the current study, we performed a sensitivity analysis. Participants with less than two years of follow-up were excluded from the principal component analysis.

Statistical analyses were conducted using R software, version 4.1.3. All P-values were 2-sided and a P < 0.05 was considered statistically significant unless otherwise stated.

## 3 Result

### 3.1 Baseline characteristics of study participants

**Table 1** shows baseline characteristics for all participants. The baseline data were analyzed for 69,068 older participants (median age 65 years [interquartile range 61-71]). During the 400,094 person-year follow-up period (median follow-up time 5.8 years [interquartile range 3.0-9.12]), 13664 deaths were recorded, of which 7045 were due to CVD. During the follow-up period, individuals who died were more likely to be male, had no spouse, were less physically active, were former or current smokers, drank alcohol daily, had lower weight, WC, BMI, TyG index, TC, TG, LDL-C, and higher RHR, SBP, DBP, HDL-C, and were more likely to have hypertension and T2DM. Similarly, individuals who died from CVD had similar baseline characteristics. In addition, according to the TyG index, LDL-C, and HDL-C, there were significant differences between the four groups in terms of age, gender, marital status, WC, BMI, smoking, alcohol consumption, physical activity, TC, TG, hypertension, and T2DM (**Supplementary Tables S1-S3**).

**Table 1:** Baseline characteristics of the study population stratified by outcome.

Variables	All-cause mortality		*P* value	Cardiovascular disease mortality		*P* value
	No(n=55,547)	Yes(n=13,521)		No(n=23,569)	Yes(n=6,975)	
Age, years	65.61 (5.66)	73.29 (7.93)	< 0.001	66.70 (5.79)	73.11 (7.46)	< 0.001
Gender, %			< 0.001			< 0.001
Male	25781 (46.41)	7318 (54.12)		9643 (40.91)	3632 (52.07)	
Female	29766 (53.59)	6203 (45.88)		13926 (59.09)	3343 (47.93)	
Marital status, %			< 0.001			< 0.001
married	45274 (81.51)	8716 (64.46)		18428 (78.19)	4561 (65.39)	
unmarried	808 (1.45)	367 (2.71)		373 (1.22)	139 (1.99)	
widowed	9147 (16.47)	4340 (32.10)		7025 (23.00)	2236 (32.06)	
divorced	318 (0.57)	98 (0.72)		157 (0.51)	39 (0.56)	
Weight, kg	63.19 (10.10)	60.75 (10.64)	< 0.001	62.94 (10.33)	60.76 (10.76)	< 0.001
WC, cm	85.42 (9.72)	84.08 (10.36)	< 0.001	85.87 (10.05)	84.85 (10.50)	< 0.001
Physical exercise, %			< 0.001			0.033
Highly active	12479 (22.47)	2870 (21.23)		5879 (24.94)	1628 (23.34)	
Sufficiently active	2456 (4.42)	622 (4.60)		1069 (4.54)	319 (4.57)	
Insufficiently active	2828 (5.09)	841 (6.22)		1407 (5.97)	453 (6.49)	
Inactive	37784 (68.02)	9188 (67.95)		15214 (64.55)	4575 (65.59)	
Smoking, %			< 0.001			< 0.001
Never smoker	46042 (82.89)	11017 (81.48)		19677 (83.49)	5638 (80.83)	
Former smoker	1472 (2.65)	522 (3.86)		736 (3.12)	297 (4.26)	
Current smoker	8033 (14.46)	1982 (14.66)		3156 (13.39)	1040 (14.91)	
Drinking, %			< 0.001			0.02
Never	51019 (91.85)	12480 (92.30)		21675 (91.96)	6407 (91.86)	
Once in a while	2820 (5.08)	590 (4.36)		1128 (4.79)	323 (4.63)	
More than once a week	637 (1.15)	117 (0.87)		252 (1.07)	57 (0.82)	
Every day	1071 (1.93)	334 (2.47)		514 (2.18)	188 (2.70)	
RHR, beats/min	72.49 (10.65)	74.26 (13.56)	< 0.001	72.48 (9.83)	74.09 (13.20)	< 0.001
SBP, mmHg	134.06 (19.44)	137.18 (21.39)	< 0.001	135.43 (19.96)	138.26 (21.76)	< 0.001
DBP, mmHg	79.97 (10.78)	80.04 (11.38)	0.528	79.97 (11.00)	80.20 (11.66)	0.179
TC, mmol/L	4.71 [4.13, 5.35]	4.69 [4.10, 5.30]	< 0.001	4.80 [4.20, 5.43]	4.74 [4.14, 5.39]	0.001
TG, mmol/L	1.24 [0.89, 1.65]	1.20 [0.87, 1.55]	< 0.001	1.27 [0.91, 1.69]	1.22 [0.89, 1.60]	< 0.001
LDL-C, mmol/L	2.77 [2.26, 3.26]	2.70 [2.20, 3.20]	< 0.001	2.90 [2.33, 3.40]	2.62 [2.15, 3.10]	< 0.001
HDL-C, mmol/L	1.31 [1.10, 1.60]	1.32 [1.10, 1.63]	< 0.001	1.30 [1.08, 1.59]	1.31 [1.09, 1.62]	0.002
BMI, kg/m^2^	24.79 (5.36)	24.08 (3.51)	< 0.001	24.95 (3.46)	24.25 (3.59)	< 0.001
TyG index	8.58 (0.61)	8.55 (0.62)	< 0.001	8.61 (0.61)	8.58 (0.62)	< 0.001
Hypertension, %	33021 (59.45)	8981 (66.42)	< 0.001	15910 (67.50)	4941 (70.84)	< 0.001
T2DM, %	12535 (22.57)	3467 (25.64)	< 0.001	6244 (26.49)	1948 (27.93)	0.018

Data are presented as number (percentage), mean (SD), or median [interquartile range].

BMI, body mass index; DBP, diastolic blood pressure; HDL-C, high-density lipoprotein cholesterol; LDL-C, low-density lipoprotein cholesterol; RHR, resting heart rate; SBP, systolic blood pressure; TC, total cholesterol; TG, triglyceride; TyG, triglyceride glucose; T2DM, type 2 diabetes mellitus; WC, Waist circumference.

### 3.2 Association of TyG index and LDL-C or HDL-C with all-cause and CVD mortality

After adjusting for covariates such as age, gender, marital status, physical activity, smoking, alcohol consumption, BMI, WC, hypertension, and T2DM, Cox proportional risk analyses showed that the multivariate-adjusted HR (95% CI) for all-cause mortality in the first, third, and fourth quartiles of the TyG index compared with the second quartile were 1.03 (0.99,1.09), 1.05 (1.01,1.10), and 1.16 (1.12,1.22), and the multivariable-adjusted HR (95% CI) for CVD mortality was 1.13 (1.06,1.21), 1.02 (0.96,1.09), and 1.08 (1.01,1.16), respectively.

At the same time, the risk of all-cause and CVD mortality increased significantly with increasing quartiles of LDL-C and HDL-C. Compared with the third quartile of the LDL-C, the multivariable-adjusted HR (95% CI) for all-cause mortality in the first, second, and fourth quartiles of LDL-C was 0.96 (0.92,1.01), 0.99 (0.94,1.03), and 1.07 (1.02,1.12), and that for CVD mortality was were 0.94 (0.88,1.00), 0.96 (0.90,1.03), and 1.09 (1.01,1.17), respectively. Compared with the second quartile of the HDL-C, the multivariable-adjusted HR (95% CI) for all-cause mortality in the first, third, and fourth quartiles of HDL-C was 0.97 (0.93,1.02), 1.01 (0.97,1.06), and 1.10 (1.05,1.16), and that for CVD mortality was were 0.95 (0.89,1.01), 1.07 (1.01,1.15), and 1.11 (1.04,1.18), respectively. In models 2 and 3, LDL-C and HDL-C were associated with an increasing trend in all-cause and CVD mortality (**Table 2**, *P* for trend all <0.001).

**Table 2:** Risk of all-cause and CVD mortality according to quartiles of TyG index, LDL-C and HDL-C.

Outcomes	Variables		No. of deaths	HR (95% CI)		
				Model 1	Model 2	Model 3
All-cause mortality	TyG index	Q1	3500	1.01 (0.96,1.06)	1.04 (0.99,1.09)	1.03 (0.99,1.09)
		Q2	3322	1.00 (ref)	1.00 (ref)	1.00 (ref)
		Q3	3558	1.01 (0.96,1.06)	1.06 (1.01,1.11)	1.05 (1.01,1.10)
		Q4	3141	1.01 (0.96,1.06)	1.17 (1.12,1.23)	1.16 (1.12,1.22)
	*P* for trend			0.99	< 0.001	< 0.001
	LDL-C	Q1	3347	1.07 (1.02,1.12)	0.95 (0.91,0.99)	0.96 (0.92,1.01)
		Q2	3696	1.00 (0.95,1.05)	0.98 (0.93,1.03)	0.99 (0.94,1.03)
		Q3	3462	1.00 (ref)	1.00 (ref)	1.00 (ref)
		Q4	3016	1.01 (0.96,1.06)	1.08 (1.03,1.14)	1.07 (1.02,1.12)
	*P* for trend			0.02	< 0.001	< 0.001
	HDL-C	Q1	3297	1.02 (0.97,1.07)	0.98 (0.94,1.03)	0.97 (0.93,1.02)
		Q2	3307	1.00 (ref)	1.00 (ref)	1.00 (ref)
		Q3	3330	1.00 (0.96,1.05)	1.01 (0.97,1.06)	1.01 (0.97,1.06)
		Q4	3587	1.08 (1.03,1.13)	1.10 (1.05,1.15)	1.10 (1.05,1.16)
	*P* for trend			0.003	< 0.001	< 0.001
Cardiovascular disease mortality	TyG index	Q1	1715	1.13 (1.06,1.21)	1.14 (1.07,1.22)	1.13 (1.06,1.21)
		Q2	1714	1.00 (ref)	1.00 (ref)	1.00 (ref)
		Q3	1783	0.96 (0.90,1.02)	1.03 (0.96,1.10)	1.02 (0.96,1.09)
		Q4	1763	0.98 (0.92,1.05)	1.07 (1.01,1.15)	1.08 (1.01,1.16)
	*P* for trend			< 0.001	< 0.001	< 0.001
	LDL-C	Q1	2088	1.01 (0.94,1.07)	0.94 (0.88,1.00)	0.94 (0.88,1.00)
		Q2	1845	0.99 (0.92,1.06)	0.96 (0.90,1.03)	0.96 (0.90,1.03)
		Q3	1642	1.00 (ref)	1.00 (ref)	1.00 (ref)
		Q4	1400	1.04 (0.97,1.12)	1.09 (1.02,1.18)	1.09 (1.01,1.17)
	*P* for trend			0.5	< 0.001	< 0.001
	HDL-C	Q1	1794	0.98 (0.91,1.04)	0.95 (0.89,1.01)	0.95 (0.89,1.01)
		Q2	1684	1.00 (ref)	1.00 (ref)	1.00 (ref)
		Q3	1705	1.08 (1.01,1.16)	1.07 (1.01,1.15)	1.07 (1.01,1.15)
		Q4	1792	1.14 (1.07,1.22)	1.11 (1.04,1.18)	1.11 (1.04,1.18)
	*P* for trend			< 0.001	< 0.001	< 0.001

TyG index: Q1 (3.19-8.19), Q2 (8.19-8.55), Q3 (8.55-8.90), Q4(8.90-13.51). LDL-C: Q1 (0.04-2.25), Q2 (2.25-2.76), Q3 (2.76-3.26), Q4(3.26-12.10). HDL-C: Q1 (0.02-1.10), Q2 (1.10-1.31), Q3 (1.31-1.61), Q4(1.61-11.68).

Model 1: Unadjusted.

Model 2: Adjusted for gender and age.

Model 3: Adjusted for gender, age, marital status, current smoking, alcohol consumption, T2DM, SBP, DBP, RHR, WC, and BMI.

HR, hazards ratio; CI, confidence interval.

**Figure 2:**
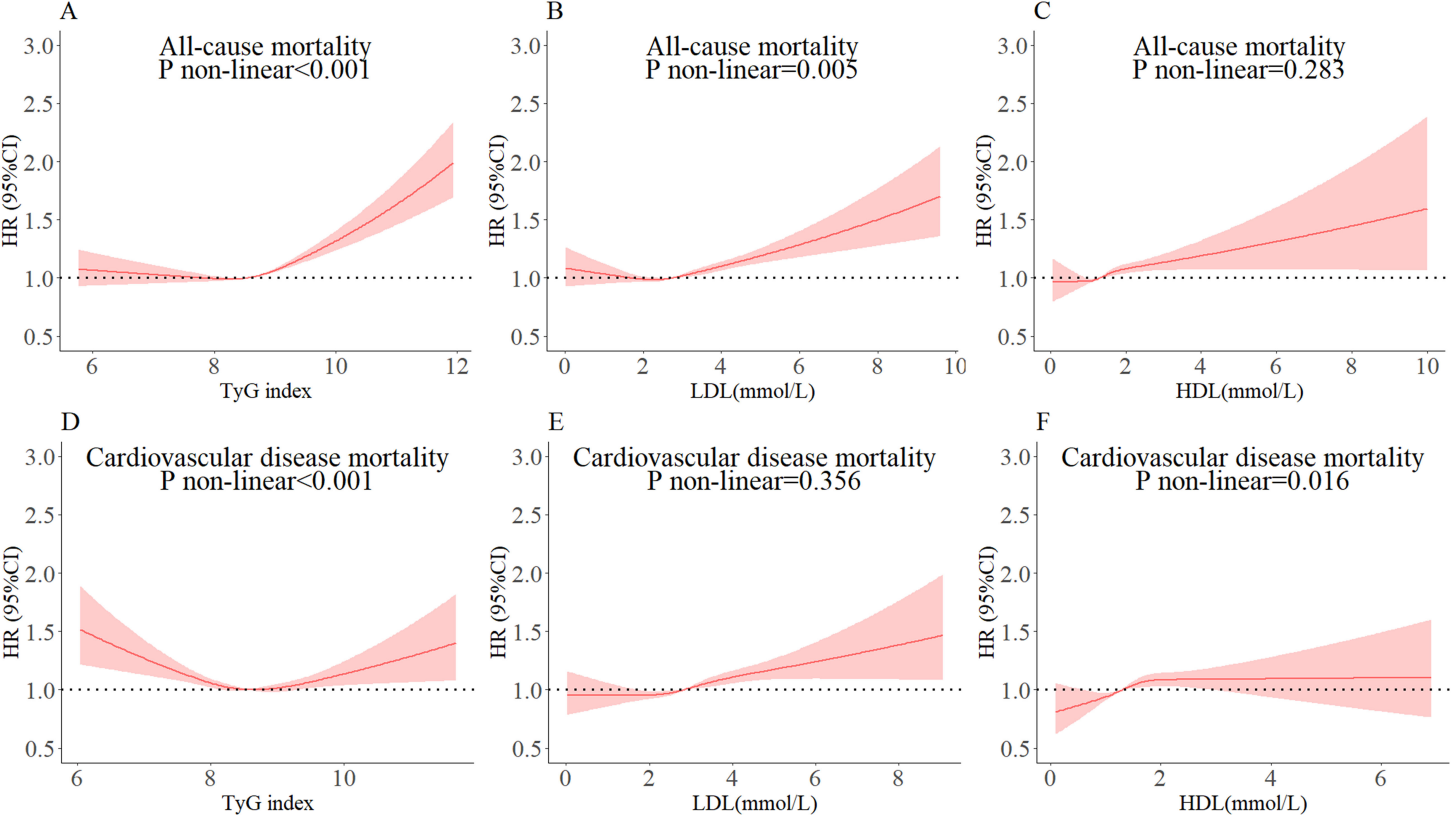
Dose-response relationships of TyG index, LDL-C, and HDL-C with all-cause and cardiovascular mortality. The cut-off levels for TyG index in all-cause and cardiovascular mortality were 8.56 **(A)** and 8.74 **(D)**, for LDL-C 1.66 **(B)** and 2.84 **(E)**, and for HDL-C 1.36 **(C)** and 1.30 **(F)**, respectively. The circles represent the points (5, 25, 50, 75, and 95 percentiles) where the nodes were placed. The region between the two dotted lines represents the 95% confidence interval (95% CI). The model was adjusted for gender, age, marital status, current smoking, alcohol consumption, T2DM, SBP, DBP, RHR, WC, and BMI.

Cox proportional risk regression models with restricted cubic spline were used to estimate the dose-response relationships of TyG index, LDL-C, and HDL-C with all-cause and CVD mortality. The three nodes of the cubic spline curve were set at the 10th, 50th, and 90th percentiles, respectively. The results showed non-linear associations between TyG index and all-cause (**Figure 2A**, *P*_non-linear_ <0.001) and CVD mortality (**Figure 2D**, *P*_non-linear_ <0.001) after adjusting for covariates in model 3. The study found J-shaped associations between both LDL-C and all-cause mortality (**Figure 2B**, *P*_non-linear_ <0.05) and HDL-C and CVD mortality (**Figure 2F**, *P*_non-linear_ <0.05). However, no non-linear associations were found between LDL-C and all-cause mortality, and HDL-C and CVD mortality (**Figures 2C, E**, all *P*_non-linear_ >0.05). It is important to note that the TyG index and CVD mortality had a U-shaped association.

The inflection points of TyG index, LDL-C, or HDL-C for all-cause and CVD mortality were determined using a two-segment Cox proportional risk-based regression model (**Table 3**). After adjusting for covariates in model 3, both TyG index, LDL-C, and HDL-C were found to be significantly and positively associated with all-cause and CVD mortality when above the inflection point. It should be noted that CVD mortality decreased by 5% when the TyG index was below the inflection point (HR: 0.95, 95% CI: 0.92, 0.98). Additionally, when LDL-C was below the inflection point, all-cause mortality decreased by 22% (HR: 0.78, 95% CI: 0.64, 0.96).

**Table 3:** Threshold effect analysis of TyG index, LDL-C, and HDL-C on all-cause and CVD mortality.

Outcome	Variables	Inflection point	HR (95% CI)	*P*-value
All-cause mortality	TyG index	8.56		
	<8.56		0.97 (0.92,1.04)	0.401
	≥8.56		1.21 (1.15,1.28)	< 0.001
	LDL-C	1.66		
	<1.66		0.78 (0.64,0.96)	0.016
	≥1.66		1.08 (1.05,1.11)	< 0.001
	HDL-C	1.36		
	<1.36		1.10 (0.97,1.25)	0.140
	≥1.36		1.07 (1.02,1.11)	0.002
Cardiovascular disease mortality	TyG index	8.74		
	<8.74		0.95 (0.92,0.98)	< 0.001
	≥8.74		1.05 (1.01,1.09)	0.02
	LDL-C	2.84		
	<2.84		1.01 (0.98,1.04)	0.458
	≥2.84		1.07 (1.03,1.11)	0.002
	HDL-C	1.30		
	<1.30		1.03 (0.99,1.06)	0.08
	≥1.30		1.03 (0.97,1.09)	0.336

The lowest point of the continuous variables TyG index, LDL-C, and HDL-C mortality risk ratios was taken as the cutoff value, and the mortality risk ratios for each variable on both sides were calculated at the cutoff value, respectively. Model was adjusted for gender, age, marital status, current smoking, alcohol consumption, T2DM, SBP, DBP, RHR, WC, and BMI. Abbreviations: HR, hazards ratio; CI, confidence interval.

To examine the relationship between the TyG index and LDL-C or HDL-C and the risk of all-cause and CVD mortality, we divided the participants into four subgroups based on their baseline TyG index levels and whether their LDL-C or HDL-C levels were within the normal range (**Table 4**). Compared to the low TyG index combined with LDL-C normal group, participants in the high TyG index combined with LDL-C abnormal group had a 14% (adjusted HR: 1.14, 95% CI: 1.08,1.20), and 13% (adjusted HR: 1.13, 95% CI: 1.04,1.22) increase in all-cause and CVD mortality, respectively. No significant interactive effects of TyG index and LDL-C on the risk of all-cause and CVD mortality. Participants in the high TyG index combined with the abnormal HDL-C group had an 11% increase in all-cause mortality compared to the low TyG index combined with the normal HDL-C group (adjusted HR: 1.11, 95% CI: 1.04,1.17). However, a high TyG index combined with HDL-C abnormality did not significantly increase CVD mortality. Similarly, there were no significant interactive effects of TyG index and HDL-C on the risk of all-cause and CVD mortality.

**Table 4:** HR of all-cause and CVD mortality by combined categories of TyG index, LDL-C, and HDL-C.

Outcomes	TyG index	LDL-C		*P*-interaction	HDL-C		*P*-interaction
		normal	abnormal		normal	abnormal	
All-cause mortality	TyG index < 8.56	1.00 (ref)	1.10 (1.05,1.17)	0.021	1.00 (ref)	1.09 (1.03,1.15)	0.036
	TyG index ≥ 8.56	1.09 (1.05,1.14)	1.14 (1.08,1.20)		1.10 (1.05,1.14)	1.11 (1.04,1.17)	
Cardiovascular disease mortality	TyG index< 8.74	1.00 (ref)	1.11 (1.04,1.19)	0.041	1.00 (ref)	0.96 (0.89,1.03)	0.129
	TyG index≥ 8.74	1.01 (0.95,1.07)	1.13 (1.04,1.22)		1.01 (0.95,1.07)	1.00 (0.91,1.09)	

The relationship between normal and abnormal LDL-C or HDL-C and the risk of mortality under these conditions was determined by calculating the interaction using the cutoff value of the TyG index as the cutoff value for categorization into high and low TyG index, respectively. Data are presented as hazard ratios, 95% confidence intervals. Model was adjusted for gender, age, marital status, current smoking, alcohol consumption, T2DM, SBP, DBP, RHR, WC, and BMI.

### 3.3 Subgroup analyses and sensitivity analyses

**Figure 3:**
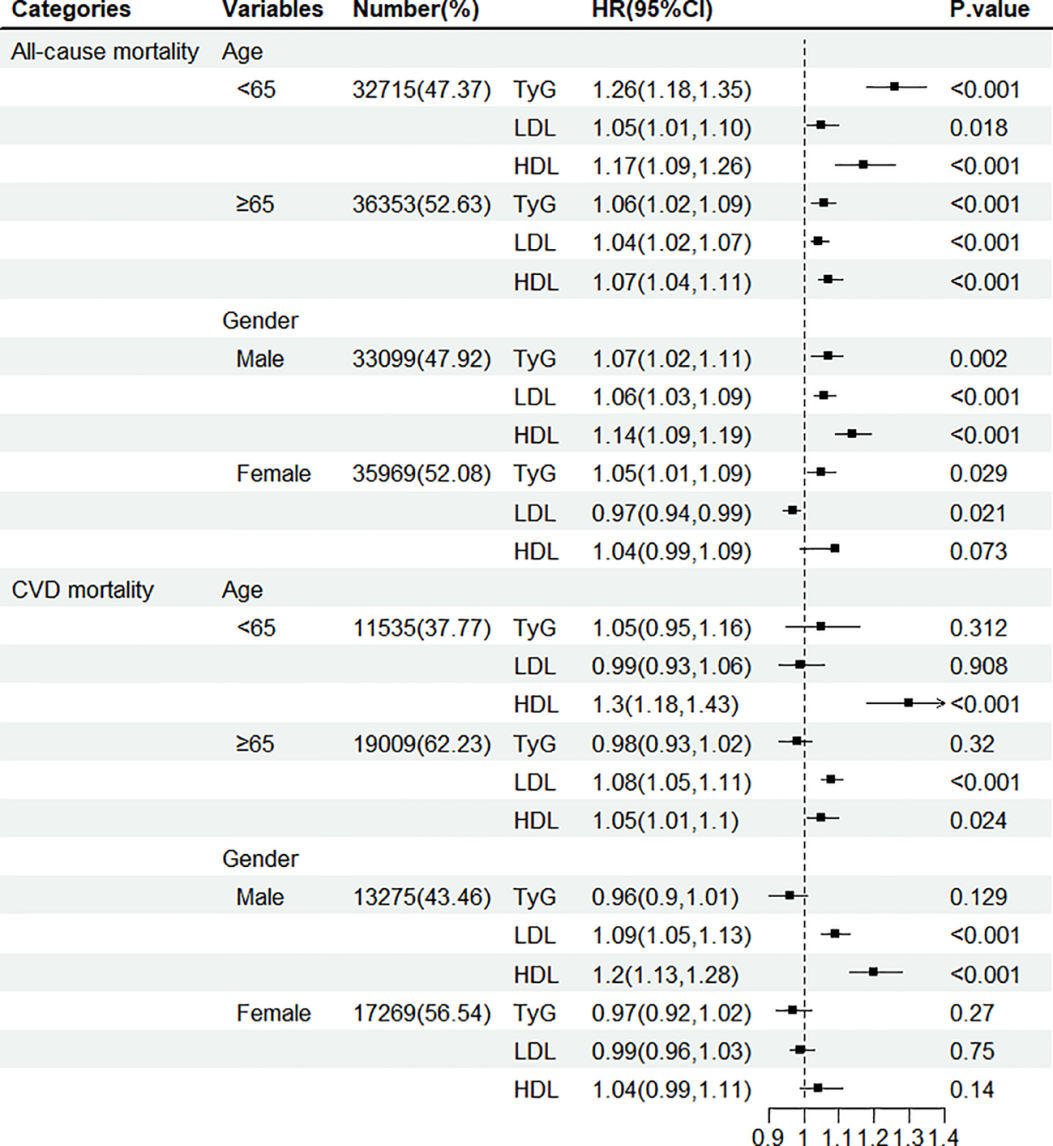
Hazard ratio (95% confidence interval) of all-cause and CVD mortality for per 1-SD increase in TyG index, LDL-C, and HDL-C according to gender and age.

Subgroup analyses showed that TyG index and LDL-C or HDL-C were more consistently positively associated with all-cause mortality by gender, and age (**Figure 3**). Sensitivity analyses produced results consistent with the main analysis (**Supplementary Table S4**, **Supplementary Figure S1**).

## 4 Discussion

In this study, we found that after adjusting for various covariates, the TyG index had a non-linear association with the risk of all-cause and CVD mortality. LDL-C had a non-linear association with the risk of all-cause mortality and a linear with CVD mortality. HDL-C had a linear association with the risk of all-cause mortality and a non-linear with CVD mortality. Additionally, through threshold effect analysis, we have identified a turning point for the TyG index (8.56 for all-cause mortality and 8.74 for CVD mortality). At this point, a high TyG index combined with abnormal LDL-C significantly increases the risk of all-cause and CVD mortality. Furthermore, the combination of abnormal HDL-C significantly increases the risk of all-cause mortality only. It is noteworthy that the fourth quartile of the TyG index is associated with lower mortality than the third quartile. This finding is inconsistent with the j-shaped curve observed in the restricted cubic spline plot. This apparent incongruity may have arisen because the value of the vertical coordinate of the restricted cubic spline plot plotted under the survival analysis condition is the risk ratio, whereas the number of outcome events presented in the table represents only one of the two variables of the survival analysis, i.e., it is the survival outcome. However, in the context of survival analysis, it is essential to consider the survival time of the subjects. The data demonstrated that individuals who died in the fourth quartile of the TyG index survived for a significantly longer period than those who died in the other three quartiles. Although the risk ratio increased in the fourth quartile group, the magnitude was less pronounced, suggesting that fewer individuals died in the fourth quartile group than in the third quartile group.

Some aspects of our findings are consistent with previous studies that have shown a positive correlation between higher TyG index and higher all-cause and CVD mortality. For instance, a cohort study conducted by the National Health and Nutrition Examination Survey, which included 20,194 participants and had a follow-up period of 9.82 years, demonstrated a non-linear relationship between the TyG index and all-cause and CVD mortality in the general population. The study found that the lowest risk of all-cause or CVD mortality occurred when the TyG index was 9.36 or 9.52 (29). Another recent study, which only included adults 18 years of age and older, similarly demonstrated a non-linear association between the TyG index and all-cause and CVD mortality. However, this study found a shift from a non-linear association to a linear positive association between the TyG index and CVD mortality when focusing on study participants aged 45-64 years (30). One possible explanation for the association between age and the TyG index is that younger people are believed to be more susceptible to IR (31), which is closely linked to the TyG index (32). As a result, as the TyG index increases, this group is more likely to develop concomitant metabolic diseases that contribute to CVD mortality. Additionally, studies investigating the relationship between the TyG index and cardiovascular metabolic multimorbidity (CMM) have discovered U-shaped associations between the TyG index and all-cause and CVD mortality (33). However, conflicting results exist regarding the effect of the TyG index on all-cause and CVD mortality in older adults. A meta-analysis that included 12 cohort studies found no statistical correlation between the TyG index and all-cause and CVD mortality (34). This lack of correlation may be due to the small number of studies included in the analysis.

Previous studies have found a strong association between lower or higher elevations of FPG and CVD morbidity and mortality even in nondiabetic patients, with a J-shaped association between blood glucose levels and CVD mortality (35). A study of TG reported that elevated TG was associated with a reduced risk of CVD mortality (36). Low TG and blood glucose may represent individuals in a poorer nutritional state, and in addition, hypoglycemia-induced thrombosis contributes to increased CVD mortality (37). Therefore, we need to maintain normal TyG index levels.

A cohort study in Denmark found that higher LDL-C was associated with an increased risk of all-cause and CVD mortality (18). Similarly, a meta-analysis of 14 studies found that LDL-C is associated with a higher risk of CVD mortality (38). These findings support the view that LDL-C is the ‘bad cholesterol’ and that higher levels of LDL-C are associated with an increased risk of death (14). Our study is similar to these studies and similarly demonstrates the association of higher LDL-C with death from CVD. Also, the fact that LDL-C collection preceded and the outcome of death appeared later in the study population confirms the causal relationship between LDL-C and CVD mortality. Regarding the evidence for HDL-C, it was found that higher levels of HDL-C were associated with an increased risk of all-cause mortality (39). This is consistent with the findings of a cohort study conducted in Copenhagen, where HDL-C is known as an ‘anti-atherogenic lipoprotein’ that prevents atherosclerosis and slows the onset of CVD (40). However, our study found the opposite. Individuals who died of all-cause and cardiovascular disease during the follow-up period were, on average, approximately seven to eight years older than those who survived. However, individuals with lower high-density lipoprotein cholesterol (HDL-C) may have died during the 5.8-year follow-up period and therefore could not be included in the study. Due to the survival effect, high-density lipoprotein cholesterol (HDL-C) was observed to be higher in the deceased cohort than in the surviving cohort. Consequently, it is plausible that elevated HDL-C levels may be associated with an increased risk of all-cause mortality.

In this study, we analyzed the interaction between the TyG index and LDL-C or HDL-C about the risk of all-cause and CVD mortality. Our findings suggest that an elevated TyG index is more strongly associated with all-cause and CVD mortality in subjects with abnormal LDL-C, and with all-cause mortality in those with abnormal HDL-C. It is important to note that the TyG index and serum cholesterol level are two independent measurements. However, the TyG index is obtained from the combined measurement of TG and FPG. TG and LDL-C or HDL-C levels are often considered interrelated biomarkers of the underlying state of the circulatory and cardiovascular systems. Hypertension, hyperlipidemia, and hyperglycemia have been referred to as the ‘three highs.’ Studies have demonstrated that they interact with each other (41). It is worth mentioning that previous researchers have used the ratio of TG to HDL-C in combination with the TyG index to explore associations with the development of CVD (42). The potential biological mechanisms through which the TyG index and LDL-C or HDL-C are linked to all-cause mortality and CVD mortality, respectively, are outlined below. A reduction in NO results in impaired endothelial-dependent vasodilation. Moreover, impaired vasodilation is not a cause or risk of diabetes; on the contrary, endothelial dysfunction is a frequent consequence of diabetes (43, 44). Furthermore, evidence indicates that IR serves as a marker for cardiovascular metabolic diseases (CMD) and dyslipidemia (45). Elevated blood lipids have been demonstrated to promote the formation of atherosclerotic plaques (46). Furthermore, dyslipidemia is associated with an increased risk of thrombotic events and, consequently, mortality from cardiovascular causes (47). The aforementioned mechanisms may partially elucidate the potential correlation between the TyG index and lipids and the risk of mortality in patients with cardiovascular disease (48). There was no consistency found between the TyG index and LDL-C or HDL-C level to all-cause mortality across different age and sex groups. The effect of the TyG index and LDL-C or HDL-C level on all-cause mortality decreased with age among females. However, a positive correlation still reached a significant level even among those over 65 years of age, which is consistent with previous findings (49–51). It is worth noting that the risk of all-cause mortality was greater for each 1 SD increase in HDL-C in participants younger than 65 years of age compared with participants older than 65 years of age. This suggests that older adults, especially those in the early stages of aging, should pay particular attention to HDL-C metrics. The reason for this phenomenon may be related to the fact that the participants were in a degenerative stage of body functions when they first entered old age.

The study has several strengths. Firstly, the data were obtained from the records of annual health checkups in Xinzheng City, Henan Province, China, focusing on adults aged 60 years and older, which represents a large sample size. Secondly, instruments were used to measure participants’ serum cholesterol levels rather than relying on self-reporting, reducing information bias. Additionally, our mortality data were obtained from the Centers for Disease Control and Prevention, and the causes of death were reviewed by at least three clinical experts. Finally, no previous study has explored the interaction between the TyG index and serum cholesterol levels, and we explored whether there was an interaction between the two. However, this study has some limitations. Firstly, individuals who died of all-cause and CVD during the follow-up period were approximately 7 to 8 years older than those who did not die. However, during the follow-up time of 5.8 years, some of their peers with higher TyG index, LDL-C, and lower HDL-C may have died, and thus they could not be recruited into the study. Due to survival effects, the TyG index and LDL-C were lower in those who died than in those who did not die, and HDL-C was higher than in those who died. It is therefore possible that the data presented in the study may be biased. Secondly, the latent category trajectory model (LCGM) analysis highlights the importance of considering dynamic changes in participant measures during follow-up, regardless of baseline. Furthermore, the questionnaire did not include information on diet and cardiorespiratory fitness, which would have helped to eliminate the possibility of reverse causation and residual confounding. Additionally, the study focused on individuals aged 60 years and older, which may limit generalizability to the entire population.

## 5 Conclusion

In this study, we found that TyG index and LDL-C or HDL-C were significantly associated with an increased risk of all-cause and CVD mortality in a Chinese population of older adults. Furthermore, a high TyG index combined with abnormal LDL-C levels was also associated with an elevated risk. These findings suggest that routine monitoring and control of TyG index and lipids should be strengthened, and these indices should be included in risk assessment as risk factors for all-cause and CVD mortality.

## Data Availability

The original contributions presented in the study are included in the article/**Supplementary Material**. Further inquiries can be directed to the corresponding author.
