# Supplementary material for: Association of triglyceride-glucose index, low and high-density lipoprotein cholesterol with all-cause and cardiovascular disease mortality in generally Chinese elderly: a retrospective cohort study
Source: Front Endocrinol (Lausanne). 2024 Oct 29;15:1422086. doi: 10.3389/fendo.2024.1422086 (PMC11554468; doi:10.3389/fendo.2024.1422086)
Supplement: Supplementary file 1 [file DataSheet1.docx]

| **Supplemental Table S1** Baseline characteristics of the study population according to TyG index | | | | | |
| --- | --- | --- | --- | --- | --- |
| Variables | TyG index | | | | *P* value |
|  | Q1(TyG<8.19) | Q2(8.19≤TyG<8.55) | Q3(8.55≤TyG<8.90) | Q4(TyG≥8.90) |  |
| n, % | 17141 | 16784 | 17832 | 17311 |  |
| Age, years | 67.15 (7.01) | 67.38 (7.10) | 67.37 (6.97) | 66.55 (6.39) | <0.001 |
| Gender, % |  |  |  |  | <0.001 |
| Male | 9894 (57.72) | 8473 (50.48) | 8076 (45.29) | 6656 (38.45) |  |
| Female | 7247 (42.28) | 8311 (49.52) | 9756 (54.71) | 10655 (61.55) |  |
| Marital status, % |  |  |  |  | <0.001 |
| married | 13375 (78.03) | 13048 (77.74) | 13848 (77.66) | 13719 (79.25) |  |
| unmarried | 411 (2.40) | 293 (1.75) | 269 (1.51) | 202 (1.17) |  |
| widowed | 3255 (18.99) | 3326 (19.82) | 3603 (20.21) | 3303 (19.08) |  |
| divorced | 100 (0.58) | 117 (0.70) | 112 (0.63) | 87 (0.50) |  |
| Weight, kg | 60.98 (9.79) | 62.03 (10.10) | 62.73 (10.17) | 65.07 (10.50) | <0.001 |
| WC, cm | 82.77 (9.20) | 84.12 (9.65) | 85.43 (9.86) | 88.26 (9.88) | <0.001 |
| Physical exercise, % |  |  |  |  | <0.001 |
| Highly active | 3211 (18.73) | 3531 (21.04) | 4045 (22.68) | 4562 (26.35) |  |
| Sufficiently active | 811 (4.73) | 705 (4.20) | 786 (4.41) | 776 (4.48) |  |
| Insufficiently active | 890 (5.19) | 901 (5.37) | 973 (5.46) | 905 (5.23) |  |
| Inactive | 12229 (71.34) | 11647 (69.39) | 12028 (67.45) | 11068 (63.94) |  |
| Smoking, % |  |  |  |  | <0.001 |
| Never smoker | 13644 (79.60) | 13777 (82.08) | 14827 (83.15) | 14811 (85.56) |  |
| Former smoker | 551 (3.21) | 521 (3.10) | 513 (2.88) | 409 (2.36) |  |
| Current smoker | 2946 (17.19) | 2486 (14.81) | 2492 (13.97) | 2091 (12.08) |  |
| Drinking, % | 15673 (91.44) | 15454 (92.08) | 16424 (92.10) | 15948 (92.13) | <0.001 |
| Never | 966 (5.64) | 838 (4.99) | 832 (4.67) | 774 (4.47) |  |
| Once in a while | 192 (1.12) | 178 (1.06) | 185 (1.04) | 199 (1.15) |  |
| More than once a week | 310 (1.81) | 314 (1.87) | 391 (2.19) | 390 (2.25) |  |
| Every day | 13644 (79.60) | 13777 (82.08) | 14827 (83.15) | 14811 (85.56) |  |
| RHR, beats | 71.82 (10.39) | 72.56 (10.50) | 72.94 (12.17) | 74.01 (11.87) | <0.001 |
| SBP, mmHg | 131.28 (18.84) | 133.52 (19.57) | 135.01 (19.69) | 138.80 (20.59) | <0.001 |
| DBP, mmHg | 78.68 (10.70) | 79.39 (10.78) | 79.97 (10.71) | 81.86 (11.17) | <0.001 |
| TC, mmol/L | 4.34 [3.76, 4.93] | 4.60 [4.08, 5.20] | 4.84 [4.29, 5.40] | 5.10 [4.45, 5.80] | <0.001 |
| TG, mmol/L | 0.70 [0.58, 0.82] | 1.07 [0.95, 1.20] | 1.40 [1.28, 1.54] | 2.12 [1.75, 2.74] | <0.001 |
| LDL-C, mmol/L | 2.62 [2.15, 3.10] | 2.72 [2.23, 3.21] | 2.80 [2.30, 3.30] | 2.90 [2.33, 3.39] | <0.001 |
| HDL-C, mmol/L | 1.36 [1.15, 1.69] | 1.32 [1.10, 1.61] | 1.30 [1.09, 1.60] | 1.26 [1.04, 1.52] | <0.001 |
| BMI ,kg/m^2^ | 23.73 (3.19) | 24.30 (3.43) | 24.76 (8.03) | 25.79 (3.45) | <0.001 |
| Hypertension, % | 8906 ( 51.96) | 9705 (57.82) | 11153 (62.54) | 12238 (70.69) | <0.001 |
| T2DM, % | 1937 ( 11.30) | 2561 (15.26) | 3860 (21.65) | 7644 (44.16) | <0.001 |
| Data are presented as number (percentage), means (SD), or median [interquartile range].  Abbreviations: BMI, body mass index; DBP, diastolic blood pressure; HDL-C, high-density lipoprotein cholesterol; LDL-C, low-density lipoprotein cholesterol; RHR, resting heart rate; SBP, systolic blood pressure; TC, total cholesterol; TG, triglyceride; TyG, triglyceride glucose; T2DM, type 2 diabetes mellitus; WC, Waist circumference. | | | | | |

| **Supplemental Table S2** Baseline characteristics of the study population according to LDL-C | | | | | |
| --- | --- | --- | --- | --- | --- |
| Variables | LDL-C | | | | *P* value |
|  | Q1(LDL-C <2.25) | Q2(2.25≤LDL-C <2.76) | Q3(2.76≤LDL-C <3.26) | Q4(LDL-C≥3.26) |  |
| n, % | 17282 | 17288 | 17439 | 17059 |  |
| Age, years | 67.29 (7.13) | 67.06 (6.87) | 67.19 (6.87) | 66.92 (6.62) | <0.001 |
| Gender, % |  |  |  |  | <0.001 |
| Male | 9657 (55.88) | 8767 (50.71) | 7849 (45.01) | 6826 (40.01) |  |
| Female | 7625 (44.12) | 8521 (49.29) | 9590 (54.99) | 10233 (59.99) |  |
| Marital status, % |  |  |  |  | <0.001 |
| married | 13471 (77.95) | 13544 (78.34) | 13585 (77.90) | 13390 (78.49) |  |
| unmarried | 280 (1.62) | 275 (1.59) | 289 (1.66) | 331 (1.94) |  |
| widowed | 3367 (19.48) | 3355 (19.41) | 3480 (19.96) | 3285 (19.26) |  |
| divorced | 164 (0.95) | 114 (0.66) | 85 (0.49) | 53 (0.31) |  |
| Weight, kg | 62.98 (10.24) | 62.69 (10.18) | 62.64 (10.27) | 62.53 (10.32) | <0.001 |
| WC, cm | 84.61 (9.93) | 84.81 (9.77) | 85.27 (9.83) | 85.95 (9.88) | <0.001 |
| Physical exercise, % |  |  |  |  | <0.001 |
| Highly active | 3326 (19.25) | 3575 (20.68) | 4058 (23.27) | 4390 (25.73) |  |
| Sufficiently active | 955 (5.53) | 847 (4.90) | 699 (4.01) | 577 (3.38) |  |
| Insufficiently active | 874 (5.06) | 893 (5.17) | 981 (5.63) | 921 (5.40) |  |
| Inactive | 12127 (70.17) | 11973 (69.26) | 11701 (67.10) | 11171 (65.48) |  |
| Smoking, % |  |  |  |  | <0.001 |
| Never smoker | 14267 (82.55) | 14340 (82.95) | 14420 (82.69) | 14032 (82.26) |  |
| Former smoker | 423 (2.45) | 383 (2.22) | 526 (3.02) | 662 (3.88) |  |
| Current smoker | 2592 (15.00) | 2565 (14.84) | 2493 (14.30) | 2365 (13.86) |  |
| Drinking, % |  |  |  |  | <0.001 |
| Never | 16013 (92.66) | 16040 (92.78) | 15987 (91.67) | 15459 (90.62) |  |
| Once in a while | 784 (4.54) | 752 (4.35) | 891 (5.11) | 983 (5.76) |  |
| More than once a week | 215 (1.24) | 181 (1.05) | 176 (1.01) | 182 (1.07) |  |
| Every day | 270 (1.56) | 315 (1.82) | 385 (2.21) | 435 (2.55) |  |
| RHR, beats | 73.09 (14.10) | 72.83 (10.98) | 72.77 (9.72) | 72.67 (9.84) | 0.005 |
| SBP, mmHg | 134.34 (19.66) | 134.40 (19.69) | 134.50 (19.92) | 135.47 (20.22) | <0.001 |
| DBP, mmHg | 80.15 (10.85) | 80.06 (10.86) | 79.79 (10.83) | 79.93 (11.06) | 0.013 |
| TC, mmol/L | 4.50 [3.89, 5.13] | 4.63 [4.10, 5.22] | 4.80 [4.21, 5.40] | 4.93 [4.30, 5.59] | <0.001 |
| TG, mmol/L | 1.18 [0.83, 1.55] | 1.21 [0.87, 1.60] | 1.26 [0.90, 1.67] | 1.30 [0.95, 1.73] | <0.001 |
| HDL-C, mmol/L | 1.28 [1.07, 1.55] | 1.30 [1.09, 1.57] | 1.30 [1.09, 1.60] | 1.36 [1.14, 1.70] | <0.001 |
| TyG index | 8.49 (0.60) | 8.54 (0.59) | 8.60 (0.62) | 8.66 (0.62) |  |
| BMI, kg/m^2^ | 24.48 (3.29) | 24.57 (8.12) | 24.71 (3.65) | 24.85 (3.47) | <0.001 |
| Hypertension, % | 9941 (57.52) | 10083 (58.32) | 10796 (61.91) | 11182 (65.55) | <0.001 |
| T2DM, % | 3556 (20.58) | 3747 (21.67) | 4229 (24.25) | 4470 (26.20) | <0.001 |
| Data are presented as number (percentage), means (SD), or median [interquartile range].  Abbreviations: BMI, body mass index; DBP, diastolic blood pressure; HDL-C, high-density lipoprotein cholesterol; LDL-C, low-density lipoprotein cholesterol; RHR, resting heart rate; SBP, systolic blood pressure; TC, total cholesterol; TG, triglyceride; TyG, triglyceride glucose; T2DM, type 2 diabetes mellitus; WC, Waist circumference. | | | | | |

| **Supplemental Table S3** Baseline characteristics of the study population according to HDL-C | | | | | |
| --- | --- | --- | --- | --- | --- |
| Variables | HDL-C | | | | *P* value |
|  | Q1(HDL-C <1.10) | Q2(1.10≤HDL-C <1.31) | Q3(1.31≤HDL-C <1.61) | Q4(HDL-C≥1.61) |  |
| n, % | 17114 | 17370 | 17395 | 17189 |  |
| Age, years | 67.28 (6.88) | 67.03 (6.84) | 66.97 (6.84) | 67.18 (6.95) | <0.001 |
| Gender, % |  |  |  |  | <0.001 |
| Male | 8660 (50.60) | 8417 (48.46) | 8353 (48.02) | 7669 (44.62) |  |
| Female | 8454 (49.40) | 8953 (51.54) | 9042 (51.98) | 9520 (55.38) |  |
| Marital status, % |  |  |  |  | <0.001 |
| married | 13226 (77.28) | 13564 (78.09) | 13739 (78.98) | 13461 (78.31) |  |
| unmarried | 319 (1.86) | 290 (1.67) | 301 (1.73) | 265 (1.54) |  |
| widowed | 3420 (19.98) | 3420 (19.69) | 3266 (18.78) | 3381 (19.67) |  |
| divorced | 149 (0.87) | 96 (0.55) | 89 (0.51) | 82 (0.48) |  |
| Weight, kg | 63.92 (10.22) | 62.99 (10.18) | 62.65 (10.15) | 61.29 (10.29) | <0.001 |
| WC, cm | 86.46 (10.03) | 85.45 (9.67) | 85.06 (9.72) | 83.68 (9.85) | <0.001 |
| Physical exercise, % |  |  |  |  | <0.001 |
| Highly active | 3690 (21.56) | 3708 (21.35) | 3881 (22.31) | 4070 (23.68) |  |
| Sufficiently active | 783 (4.58) | 745 (4.29) | 797 (4.58) | 753 (4.38) |  |
| Insufficiently active | 877 (5.12) | 932 (5.37) | 903 (5.19) | 957 (5.57) |  |
| Inactive | 11764 (68.74) | 11985 (69.00) | 11814 (67.92) | 11409 (66.37) |  |
| Smoking, % |  |  |  |  | <0.001 |
| Never smoker | 13797 (80.62) | 14329 (82.49) | 14370 (82.61) | 14563 (84.72) |  |
| Former smoker | 428 (2.50) | 502 (2.89) | 513 (2.95) | 551 (3.21) |  |
| Current smoker | 2889 (16.88) | 2539 (14.62) | 2512 (14.44) | 2075 (12.07) |  |
| Drinking, % |  |  |  |  | <0.001 |
| Never | 15597 (91.14) | 16006 (92.15) | 15994 (91.95) | 15902 (92.51) |  |
| Once in a while | 898 (5.25) | 864 (4.97) | 858 (4.93) | 790 (4.60) |  |
| More than once a week | 201 (1.17) | 171 (0.98) | 205 (1.18) | 177 (1.03) |  |
| Every day | 418 (2.44) | 329 (1.89) | 338 (1.94) | 320 (1.86) |  |
| RHR, beats | 72.88 (13.45) | 72.83 (9.68) | 72.81 (11.79) | 72.84 (9.89) | 0.937 |
| SBP, mmHg | 135.01 (19.90) | 134.64 (19.93) | 134.60 (19.73) | 134.44 (19.93) | 0.055 |
| DBP, mmHg | 79.44 (10.80) | 79.94 (10.91) | 80.06 (10.85) | 80.49 (11.02) | <0.001 |
| TC, mmol/L | 4.67 [4.08, 5.32] | 4.70 [4.13, 5.33] | 4.71 [4.14, 5.34] | 4.75 [4.16, 5.38] | <0.001 |
| TG, mmol/L | 1.30 [0.95, 1.78] | 1.25 [0.90, 1.65] | 1.21 [0.87, 1.60] | 1.18 [0.83, 1.52] | <0.001 |
| LDL-C, mmol/L | 2.68 [2.18, 3.13] | 2.72 [2.23, 3.21] | 2.74 [2.25, 3.24] | 2.90 [2.32, 3.41] | <0.001 |
| TyG index | 8.66 (0.62) | 8.58 (0.61) | 8.55 (0.61) | 8.50 (0.60) | <0.001 |
| BMI (mean (SD)) | 25.01 (3.71) | 24.70 (3.34) | 24.64 (8.12) | 24.25 (3.29) | <0.001 |
| Hypertension, % | 10575 (61.79) | 10493 (60.41) | 10531 (60.54) | 10403 (60.52) | 0.026 |
| T2DM, % | 4419 (25.82) | 4006 (23.06) | 3893 (22.38) | 3684 (21.43) | <0.001 |
| Data are presented as number (percentage), means (SD), or median [interquartile range].  Abbreviations: BMI, body mass index; DBP, diastolic blood pressure; HDL-C, high-density lipoprotein cholesterol; LDL-C, low-density lipoprotein cholesterol; RHR, resting heart rate; SBP, systolic blood pressure; TC, total cholesterol; TG, triglyceride; TyG, triglyceride glucose; T2DM, type 2 diabetes mellitus; WC, Waist circumference. | | | | | |

| **Supplemental Table S4** Risk of all-cause and CVD mortality according to quartiles of TyG index, LDL-C and HDL-C | | | | | | |
| --- | --- | --- | --- | --- | --- | --- |
| Outcomes | Variables |  | No. of deaths | HRs (95% CIs) | | |
|  |  |  |  | Model 1 | Model 2 | Model 3 |
| All-cause mortality | TyG | Q1 | 3481 | 1.01 (0.96,1.06) | 1.04 (0.99,1.09) | 1.04 (0.99,1.09) |
|  |  | Q2 | 3295 | 1.00 (ref) | 1.00 (ref) | 1.00 (ref) |
|  |  | Q3 | 3543 | 1.01 (0.97,1.06) | 1.06 (1.01,1.12) | 1.06 (1.01,1.10) |
|  |  | Q4 | 3120 | 1.01 (0.96,1.06) | 1.17 (1.12,1.23) | 1.16 (1.11,1.22) |
|  | *P* for trend | | | 0.99 | < 0.001 | < 0.001 |
|  | LDL | Q1 | 3674 | 1.06 (1.02,1.12) | 0.95 (0.91,1.00) | 0.96 (0.92,1.01) |
|  |  | Q2 | 3696 | 1.00 (0.95,1.05) | 0.98 (0.93,1.03) | 0.98 (0.94,1.03) |
|  |  | Q3 | 3442 | 1.00 (ref) | 1.00 (ref) | 1.00 (ref) |
|  |  | Q4 | 2992 | 1.01 (0.96,1.06) | 1.08 (1.03,1.13) | 1.07 (1.02,1.12) |
|  | *P* for trend | | | 0.02 | < 0.001 | < 0.001 |
|  | HDL | Q1 | 3269 | 1.02 (0.97,1.07) | 0.98 (0.93,1.03) | 0.97 (0.93,1.02) |
|  |  | Q2 | 3292 | 1.00 (ref) | 1.00 (ref) | 1.00 (ref) |
|  |  | Q3 | 3307 | 1.00 (0.95,1.05) | 1.01 (0.97,1.06) | 1.01 (0.96,1.06) |
|  |  | Q4 | 3571 | 1.08 (1.03,1.13) | 1.10 (1.05,1.15) | 1.11 (1.05,1.16) |
|  | *P* for trend | | | 0.003 | < 0.001 | < 0.001 |
| Cardiovascular disease mortality | TyG | Q1 | 1706 | 1.13 (1.06,1.21) | 1.14 (1.07,1.22) | 1.14 (1.06,1.22) |
|  |  | Q2 | 1706 | 1.00 (ref) | 1.00 (ref) | 1.00 (ref) |
|  |  | Q3 | 1775 | 0.96 (0.90,1.02) | 1.03 (0.96,1.10) | 1.03 (0.96,1.10) |
|  |  | Q4 | 1753 | 0.98 (0.92,1.05) | 1.07 (0.99,1.14) | 1.08 (1.01,1.16) |
|  | *P* for trend | | | < 0.001 | < 0.001 | < 0.001 |
|  | LDL | Q1 | 2076 | 0.99 (0.93,1.06) | 0.93 (0.87,1.00) | 0.94 (0.88,1.00) |
|  |  | Q2 | 1836 | 0.98 (0.92,1.05) | 0.96 (0.90,1.02) | 0.96 (0.90,1.03) |
|  |  | Q3 | 1639 | 1.00 (ref) | 1.00 (ref) | 1.00 (ref) |
|  |  | Q4 | 1389 | 1.03 (0.96,1.11) | 1.09 (1.01,1.17) | 1.09 (1.01,1.17) |
|  | *P* for trend | | | 0.5 | < 0.001 | < 0.001 |
|  | HDL | Q1 | 1782 | 0.97 (0.91,1.04) | 0.95 (0.89,1.01) | 0.95 (0.89,1.02) |
|  |  | Q2 | 1678 | 1.00 (ref) | 1.00 (ref) | 1.00 (ref) |
|  |  | Q3 | 1692 | 1.08 (1.01,1.16) | 1.07 (1.01,1.15) | 1.07 (1.00,1.14) |
|  |  | Q4 | 1788 | 1.14 (1.07,1.22) | 1.11 (1.04,1.19) | 1.11 (1.04,1.19) |
|  | *P* for trend | | | < 0.001 | < 0.001 | < 0.001 |
| Model 1: Unadjusted.  Model 2: Adjusted for gender and age.  Model 3: Adjusted for gender, age, marital status, current smoking, alcohol consumption, T2DM, SBP, DBP, RHR, WC, and BMI.  Abbreviations: HR, hazards ratio; CI, confidence interval. | | | | | | |

| **Supplementary Table S5** Tests of interaction for TyG index, LDL-C and HDL-C for all outcomes. | | | |
| --- | --- | --- | --- |
| Variables | Subgroup | All-cause mortality | CVD mortality |
| TyG index | Age (<65 / ≥65) | <0.001 | 0.006 |
|  | Gender (male / female) | 0.008 | 0.031 |
| LDL-C | Age (<65 / ≥65) | 0.147 | 0.023 |
|  | Gender (male / female) | 0.108 | 0.049 |
| HDL-C | Age (<65 / ≥65) | 0.305 | <0.001 |
|  | Gender (male / female) | 0.004 | <0.001 |


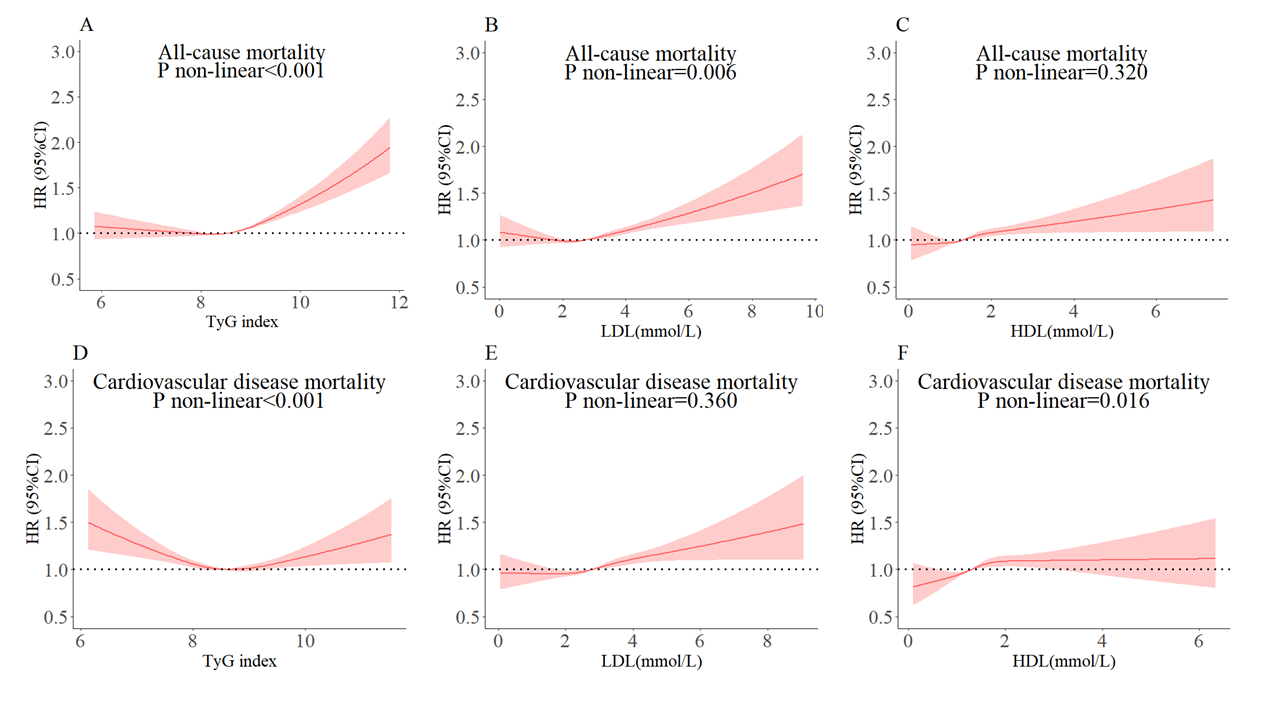


**FigureS1** Dose-response relationships of TyG index, LDL-C, and HDL-C with all-cause and cardiovascular mortality after exclude participants with less than two years of follow-up. The circles represent the points (5, 25, 50, 75, and 95 percentiles) where the nodes were placed. The region between the two dotted lines represents the 95% confidence interval (95% CI). The model was adjusted for gender, age, marital status, current smoking, alcohol consumption, T2DM, SBP, DBP, RHR, WC, and BMI.
